# DNA accessibility of chromatosomes quantified by automated image analysis of AFM data

**DOI:** 10.1038/s41598-019-49163-4

**Published:** 2019-09-04

**Authors:** Martin Würtz, Dennis Aumiller, Lina Gundelwein, Philipp Jung, Christian Schütz, Kathrin Lehmann, Katalin Tóth, Karl Rohr

**Affiliations:** 1 German Cancer Research Center, Division Biophysics of Macromolecules, Heidelberg, 69120 Germany; 2 Heidelberg University, Institute of Computer Science, Heidelberg, 69120 Germany; 3 Heidelberg University, BioQuant and IPMB, Biomedical Computer Vision Group, Heidelberg, 69120 Germany; 4 German Cancer Research Center, Heidelberg, 69120 Germany; 5 Simon Fraser University, Department of Physics, Burnaby, BC V5A 1S6 Canada

**Keywords:** Nanoscale biophysics, Single-molecule biophysics

## Abstract

DNA compaction and accessibility in eukaryotes are governed by nucleosomes and orchestrated through interactions between DNA and DNA-binding proteins. Using QuantAFM, a method for automated image analysis of atomic force microscopy (AFM) data, we performed a detailed statistical analysis of structural properties of mono-nucleosomes. QuantAFM allows fast analysis of AFM images, including image preprocessing, object segmentation, and quantification of different structural parameters to assess DNA accessibility of nucleosomes. A comparison of nucleosomes reconstituted with and without linker histone H1 quantified H1’s already described ability of compacting the nucleosome. We further employed nucleosomes bearing two charge-modifying mutations at position R81 and R88 in histone H2A (H2A R81E/R88E) to characterize DNA accessibility under destabilizing conditions. Upon H2A mutation, even in presence of H1, the DNA opening angle at the entry/exit site was increased and the DNA wrapping length around the histone core was reduced. Interestingly, a distinct opening of the less bendable DNA side was observed upon H2A mutation, indicating an enhancement of the intrinsic asymmetry of the Widom-601 nucleosomes. This study validates AFM as a technique to investigate structural parameters of nucleosomes and highlights how the DNA sequence, together with nucleosome modifications, can influence the DNA accessibility.

## Introduction

In 1974, a “beads on a string” structure was discovered in micrographs, and the spheroid unit was dubbed nucleosome^1^. Nucleosomes are the basic unit of chromatin compaction and play a key role in gene regulation. Individual nucleosomes consist of a histone octamer, which is built up by two histone H2A-H2B dimers and one histone (H3–H4)_2_ tetramer^2,3^. Around the octamer, a stretch of DNA folds around with a wrapping length of approximately 150 bp^3,4^, leading to a spool structure with a total diameter of 11 nm. The wrapping length depends on nucleosome integrity and is thus a measure for DNA accessibility. *In vivo*, DNA accessibility - and hence gene regulation - is orchestrated by many factors, for example, the presence of linker histones, post-translational modifications, or histone variants. The linker histones H1 and H5 bind to nucleosomes in the region of the DNA entry/exit site^5–11^ and can thereby increase compaction of the resulting chromatosome (nucleosome with histone H1). Early experiments with microbial nuclease digestions indicated that linker histone H1 increases the wrapping length of nucleosomes by roughly 20 bp^12^. Recent electron cryo-microscopy and crystallization studies provided detailed structural information, for example, of a 197 bp nucleosome containing the linker histone H1^6^. However, determining the exact binding configuration of the linker histone to the nucleosome is challenging since it depends on many factors like histone variants or DNA sequence^13,14^.

Post-translational modifications on histone proteins^15^ or histone variants^16–18^ have ambiguous effects on the nucleosome, since they can lead to either a more open or a more condensed structure of the chromatin. The H2A.Bbd variant, for example, preserves only 48% sequence identity to canonical H2A^19^. Nucleosomes containing H2A.Bbd were shown to have a shorter DNA wrapping length in AFM experiments, and electron microscopy (EM) revealed that the opening angle of the DNA arms was increased compared to wild type nucleosomes^20,21^. Shukla *et al*. reported an impeded binding of histone H1 to nucleosomes with H2A.Bbd variant^22^. This is just one example of the ongoing discussion about the role of histone H2A in linker histone/nucleosome interaction^10,11,22,23^.

Recently, two charge-modifying mutations in the *α*3 helices of histone H2A were shown to significantly destabilize *in vitro* reconstituted nucleosomes in a salt-induced nucleosome disassembly assay^24^. In the present study, we utilize the mutant with the most pronounced effect, namely H2A R81E/R88E^24^ as a tool to study modified chromatin in the context of DNA accessibility. The influence of the mutated histone H2A on the structural properties of chromatosomes reconstituted on the Widom-601 sequence^25^ is evaluated through a comparison with wild type chromatosomes. The complexes were measured by atomic force microscopy (AFM), since AFM^26–28^ is an established and highly suitable technique to investigate structural properties of mono-nucleosomes on a single molecule level^29,30^. Yet, evaluation of large datasets obtained with single molecule techniques is particularly important for robust statistical analysis. However, the high number of nucleosome studies where structural parameters were determined manually^24,31–34^ or image analysis was performed with a commercial (closed source) software^35–40^ indicates that open source methods for automated image analysis of nucleosome data are needed.

In this work, we present QuantAFM (Quantification of AFM images), an automated image analysis method for extraction of geometric features of filamentous structures from AFM images. QuantAFM was designed for fast conformation analysis and copes with large variation in image quality. In previous work, several algorithms for automatic extraction of DNA contour lengths were described^41–47^. Our automated method also quantifies DNA contour lengths, but additionally detects mono-nucleosomes, extracts conformational parameters like the opening angles, and allows efficient monitoring of the analyzed objects. With QuantAFM we could confirm the influence on the entry/exit DNA arms measured by the opening angle as well as the increase in DNA wrapping length upon binding of histone H1. Further, a comparison of mutated and wild type nucleosomes with and without H1 revealed that DNA accessibility is significantly enhanced (increased opening angle and decreased DNA wrapping length) for mutated nucleosomes without and with H1. Interestingly, our AFM data unravel an enhanced asymmetric nucleosome opening of the Widom-601 sequence^48–51^ upon mutation. Modification of the histone octamer, resulting in a changed electrostatic environment, can thus, depending on the DNA properties, enhance their accessibility even in the presence of histone H1.

## Results

### QuantAFM for automated image analysis of AFM data

**Figure 1:**
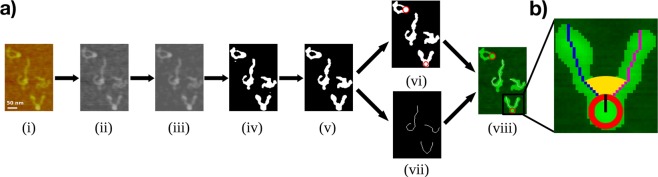
Schematic overview of the automated image analysis method and extracted parameters. (**a**) Example results illustrating the algorithmic steps of the image analysis method QuantAFM: (i) Original input image, (ii) denoising step with Non-Local Means filtering resulting in less variation of background noise^53^, (iii) additional mean and low-pass filtering for further removing the noise levels of the AFM tip, (iv) initial binarization yielding a rough segmentation result, (v) refined segmentation result after background equalization and removal of small as well as large objects, (vi) extraction of nucleosome candidates with the Hough Transform^57^ indicated by red circles, and (vii) simultaneous thinning step. (viii) Results for the thinned fragments and nucleosomes are merged based on physical proximity. Subsequently, length and angles are determined for valid fragments. (**b**) Enlarged sample nucleosome object. Determined parameters include nucleosome radius *r* (black), opening angle *θ* (yellow), and long (blue) and short (magenta) filament protruding DNA arm lengths. If no nucleosome is present, the total contour length is determined instead.

An overview of the main steps of the developed image analysis method QuantAFM is given in Fig. 1a. The general structure is similar to related work^42,43,46,47,52^ but the method includes a new combination of algorithms and improvements on certain aspects. To ensure general applicability, the input images are flattened AFM images, which do not contain further information on the height of filaments. The focus of our work was on increasing the detection rate for filaments since data acquisition is costly, and numerous of potential samples might otherwise be discarded due to suboptimal image quality. Our method extends previous methods by automated detection of mono-nucleosomes (and distinction to filaments) and quantifying opening angles, see Fig. 1b. Below, we describe the different steps of our method. For more details on the method and implementation, see Supplementary Section A.

For preprocessing of the original images, we introduce additional steps besides mean and median filtering^42,46^ to better adjust for background noise and the scan direction of the AFM tip. The latter factor results in variable contamination of the image quality with deteriorating dullness of the tip, violating the assumption of equal noise distribution within an image collection. The main performance increase was achieved by introducing a Non-Local Means Filter^53^ as a first step, and an additional low-pass filtering further helps to cope with distortions due to the scan lines of the AFM tip, see Supplementary A and Fig. 1a(ii,iii). To identify and discard image contaminations, we calculate an initial threshold based on the expected size of filaments in the data, and reset the intensities of contaminated regions to the average background intensity^46,52^, see Fig. 1a(iv). Subsequently, we recalculate the threshold based on the corrected image, which generally allowed discarding artifacts outside of the size range, compare Fig. 1a(v). Both binarization steps are performed with Otsu’s method^54^, which outperformed alternative methods for cross-dataset performance (see Supplementary Section A.3). These processing steps allow quantifying objects even in difficult images with strong contaminations and artifacts (see Supplementary Fig. S3).

After binarization, filaments are processed by thinning single strands to a backbone^42,43,47,55^, and their respective lengths are determined based on the Kulpa estimator^44,56^. To further improve the accuracy, we take the shortening caused by the thinning process into account. Results in Table 1 show high agreement with theoretical contour lengths and values obtained by manual analysis, for DNA strands of two fragment lengths, 464 bp (157.8 nm) and 599 bp (203.7 nm). Pruning of ambiguous thinning results (i.e. where a unique backbone could not be determined) was fortunately not necessary, which thus avoids discarding of objects^42,43^. Instead, we determine the maximum length based on maximum distance within the pixels of a single graph-based filament path^47^.

**Table 1:** Comparison of estimated and manual extracted contour lengths, determined for DNA of 464 bp and 599 bp.

Sample	Estimated contour length mean ± sd [nm]	Detection rate
464 bp DNA automated (*n* = 693)	158.2 ± 6.3	80%
464 bp DNA manual (*n* = 162)	157.3 ± 6.2	
599 bp DNA automated (*n* = 754)	201.0 ± 8.0	76%
599 bp DNA manual (*n* = 180)	200.9 ± 7.4	

Our image analysis method shows consistent results for both DNA lengths, as well as good detection rates for free DNA (see Supplementary Section B). Manually analyzed fragments are a subset of automated analyzed fragments.

In addition to determining the contour length, our method enables fully automated recognition of nucleosomes (Fig. 1a(vi)), which to our knowledge was not possible with previous methods. Nucleosomes have a circular shape in AFM images, attached to the filament strands. Given a radii range specified by the user, the Hough Transform^57^ is used to detect circular structures in the images. These nucleosome candidates are associated with a position in the image and a radius. They are then matched to the respective detected filaments based on their vicinity using the Euclidean distance. Associated pairs are only further evaluated if a unique match is possible (i.e. if there are not multiple nucleosome candidates attached to the same DNA strand). Beyond the nucleosome position and radius, we further determine the opening angle for wrapped DNA, and the lengths of the respective protruding DNA arms, which is measured from the intersection of strand and nucleosome. For 90% of the cases, the opening angle (*θ*) was determined correctly. For some objects the angle *θ* could not be exactly determined because of inverted angles (180° − *θ*), due to ambiguities of the orientation.

Analysis of our dataset for wt nucleosomes (599 bp) consisting of a total of 10 images with a total of 254 valid objects yielded 65% correctly recognized nucleosomes, compared to only about 15% without Non-Local Means filtering and background equalization (see Supplementary Section B). We note that results for detection without additional filters depend on the quality of the original images. Since detection rates were determined for a single threshold setting only, a higher accuracy could be achieved by iterating over different threshold values.

The focus of developing our software was on the performance and flexibility to enable application for a variety of tasks. During the runtime, QuantAFM does not require any supervision, and stores intermediate results for subsequent analysis. Even when applying our software with different sets of parameters, this is much faster than laborious manual annotation. Parameters include the enabling/disabling of several of the previously mentioned steps of the method, as well as a concise representation of filament objects and their determined parameters. QuantAFM allows easy association of extracted parameters to the respective objects in the image, enabling the user to confirm output quality of individual objects, and further accelerates supplementation of manual analysis. Importantly, we also support different resolutions both for the physical experiment, and the resulting image. Our software is implemented in MATLAB and the source code is available online.

### AFM imaging of chromatosomoes

**Figure 2:**
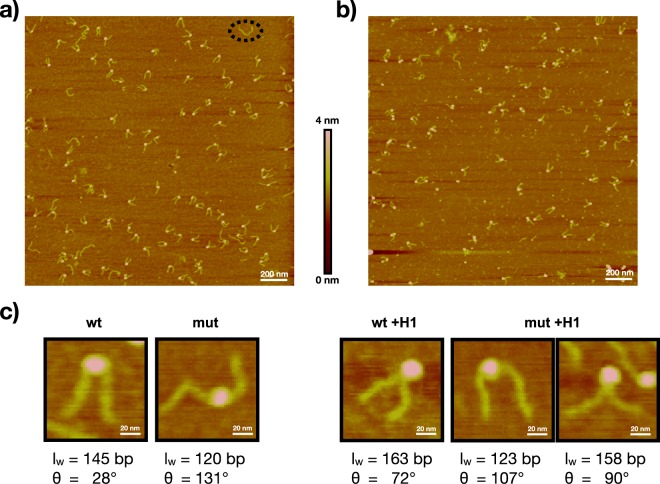
AFM imaging of chromatosomes. Nucleosome complexes deposited on PL coated surface. (**a** and **b**) Example images of wt nucleosomes reconstituted on 464 bp DNA, without and with histone H1, respectively. The images show that nucleosomes are clearly distinguishable from background and free DNA (black circle). Example images for mutated nucleosomes are shown in Supplementary Fig. S8. (**c**) Enlarged sections for each nucleosome class, selected from different images, with representative DNA wrapping length (*l*_*w*_) and trend of the opening angle *θ*. All selected complexes show a central positioning on the DNA. Mutated nucleosomes show a more open conformation. For mut +H1 a highly wrapped and less wrapped object is shown.

To demonstrate the suitability of QuantAFM for biologically relevant questions, we analyzed AFM data from wild type (wt) and H2A R81E/R88E mutated (mut^24^) mono-nucleosomes with and without linker histone H1 to investigate how DNA accessibility is affected upon mutation in H2A or histone H1 interaction. Therefore, nucleosomes were reconstituted on 464 bp long DNA containing the positioning sequence Widom-601^25^. Nucleosome reconstitution, as well as initial binding of histone H1 to both wt and mutated nucleosomes, was confirmed via electrophoretic mobility shift assay (EMSA, Supplementary Fig. S7). AFM imaging was performed via established protocols using Poly(L)lysine (PL) for surface modification^24,58,59^, where the experimental conditions^24,59^ ensure trapping of the sample on the surface^58,59^. Figure 2a,b shows representative AFM images of wt nucleosomes without and with added histone H1. Nucleosome objects are the circular objects on the filamentous DNA strands which can be clearly distinguished from free DNA molecules (Fig. 2a). Direct observation of the presence of histone H1 with our AFM setup is impeded by its resolution. Therefore, we verified that H1 binding persists upon PL deposition via surface adsorption experiments with fluorescently labeled histone H1 (Supplementary Fig. S7).

Using QuantAFM for the acquired AFM data, we quantified the influence of histone H1 on wt and especially on mutated nucleosomes to examine if histone H1 interaction can compensate the suggested destabilized behavior of the mutated nucleosomes^24^. Extracted parameters for in total $n=1,781$ nucleosome objects (Table 2) were used to compare the four classes (wt and mutated nucleosomes without and with histone H1). The determined mean values of the radius indicate that the radii of nucleosomes in the images appear, due to a AFM imaging artifact, as roughly twice the known radius. This has to be taken into consideration when determining the DNA wrapping length (*l*_*w*_), i.e. the length of DNA bound to the histones. Therefore, to calculate the *l*_*w*_ for each individual nucleosome object, we corrected the automatically determined contour lengths of the protruding DNA arms using a correction factor, and subtracted the corrected value from the mean value of the total contour length of free 464 bp DNA molecules (see Methods AFM data evaluation). The contour length of free DNA molecules was measured under the same conditions as nucleosome samples, resulting in a mean value of 158.2 nm ± 6.3 nm ($n=693$ objects), which corresponds to the expected nm/bp ratio of 0.34 ± 0.01 nm/bp.

**Table 2:** Mean values (mean) with standard deviation (sd) of extracted structural parameters of automatically analyzed AFM images from 464 bp nucleosome objects.

Parameter	Opening angle mean ± sd [°]	Radius mean ± sd [nm]	Short DNA arm mean ± sd [nm]	Long DNA arm mean ± sd [nm]
wt 464 (*n* = 444)	74.2 ± 39.6	10.6 ± 1.6	44.9 ± 6.0	53.6 ± 6.4
wt +H1 464 (*n* = 445)	75.9 ± 35.3	10.7 ± 1.6	42.4 ± 6.0	49.9 ± 5.9
mut 464 (*n* = 444)	112.6 ± 45.3	10.6 ± 1.7	47.3 ± 7.3	59.6 ± 7.8
mut +H1 464 (*n* = 448)	103.4 ± 46.2	10.2 ± 1.5	43.8 ± 7.4	56.3 ± 8.1

To illustrate the structural differences between the classes (Table 2) regarding contour lengths of the protruding DNA arms and DNA opening angle (*θ*), Fig. 2c presents for each class an individual object representing the mean characteristics of its class. The selected objects show central positioning on the DNA for all classes indicating a reconstitution on the Widom-601 sequence, and that mutated nucleosomes have a more open conformation compared to wt. The wild type chromatosome (wt +H1) shows an increased *l*_*w*_ compared to wt without histone H1. For mutated chromatosomes (mut +H1), two objects were selected underlining that within classes the complexes display large heterogeneity, which was also observed in other AFM studies with nucleosomes^21,40^. Nevertheless, on the basis of a statistically relevant number of analyzed objects a detailed comparative analysis is possible as performed in this work. In the following, we describe a comparison of the distributions of the DNA opening angle, the DNA wrapping length and the nucleosome positioning, which underpins the characteristic features of each class presented in Fig. 2c.

### Binding of histone H1 affects DNA opening angle and wrapping length

**Figure 3:**
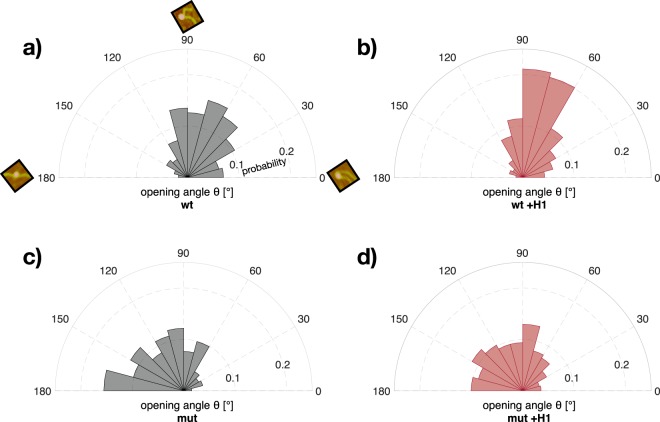
DNA opening angles are affected by mutation and histone H1 interaction. Normalized probabilities of the opening angles *θ* were plotted in 15° binned sectors for the different cases. The small example images in (**a**) represent nucleosome objects with the corresponding DNA opening angle. For each case, (**a**) wt, (**b**) wt +H1, (**c**) mut and (**d**) mut +H1 all opening angles are plotted (*n*_wt_ = 444, *n*_wt +H1_ = 445, *n*_mut_ = 444, *n*_mut +H1_ = 448). Diagrams for samples with histone H1 are shown in red. Linker histone H1 induces a distinct population between 60° and 90° in the distribution of the opening angle for wt. Compared to wt, mutated nucleosomes show higher opening angles.

A major improvement of our image analysis method compared to previous work is that the opening angle *θ* of the nucleosomes is quantified automatically. Figure 3 illustrates the distribution of *θ* for the different classes and statistical analysis was performed as described in Methods (Section Statistical data analysis). For wt nucleosomes about 70% of the opening angles are below 90° (Fig. 3a), while for mutated nucleosomes about 70% of the opening angles are above 90° (Fig. 3c). This means a significant shift towards a more open structure of the mutated nucleosome ($p\ll 0.05$). Similar observations were made for H2A.Bdb containing nucleosomes^20^.

The distribution of wt with linker histone H1 does not differ significantly from the distribution without H1, but H1 induces a preferred *θ* population between 60° and 90° resulting in a distinct DNA entry/exit site conformation (see Fig. 2c). Although for mutated nucleosomes with linker histone H1 (Fig. 3d) the majority of objects are in a more open structure with higher values of *θ*, the frequency of lower values of *θ* around 90° is also increased compared to mutated nucleosomes without H1 ($p=0.019$). The evident difference for the opening angles between wt and mutated nucleosomes underlines the impact of the charge-modifying mutations on the nucleosome conformation also in the presence of linker histone H1.

**Figure 4:**
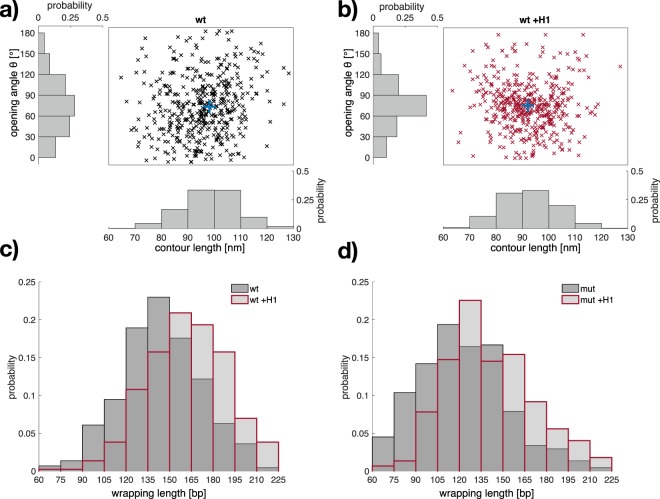
Linker histone affects both DNA wrapping length and opening angle. Top: Scatter plots of the opening angle *θ* and protruding DNA arm contour length of wt nucleosomes (**a**) without (*n*_wt_ = 444, black) and (**b**) with histone H1 (*n*_wt +H1_ = 445, red); centroids are highlighted in blue. Scatter plots for mutated nucleosomes are shown in Supplementary Fig. S8. The histograms show the normalized probabilities for the opening angle (30° bin width) and the protruding DNA arm contour length (bin width 10 nm). Histone H1 induced a decreased protruding DNA length together with a preference of a distinct opening angle. Bottom: The protruding DNA arm contour length was converted into the wrapping length for (**c**) wt nucleosome objects and (**d**) mutated nucleosome objects without and with histone H1 (*n*_wt_ = 444, *n*_wt +H1_ = 445, *n*_mut_ = 444, *n*_mut +H1_ = 448). Normalized histograms of wrapping length are plotted from 60 bp–225 bp (bin width 15 bp). Bars of samples with linker histone H1 are indicated by red edges. DNA wrapping is lower for mutated nucleosomes than wt, but increased in the presence of histone H1 in both cases.

Since our method does not only determine the opening angles but also the relevant contour length parameters of the objects, a multifaceted analysis is feasible. To emphasize the heterogeneity in object conformation, scatter plots of the opening angle versus contour length of the protruding DNA arms for wt without and with histone H1, respectively, are shown in Fig. 4a,b (scatter plots for mutated nucleosomes are shown in Supplementary Fig. S8). Upon histone H1 binding (Fig. 4b) the heterogeneity decreases with more pronounced clustering of the distribution between 60°–90° opening angle and 80 nm–100 nm protruding DNA arm contour length. As discussed for Fig. 3c, this clustering of the opening angle indicates a preferred conformation of the DNA arms at the entry/exit site. Moreover, compared to wt without H1 (Fig. 4a), the scatter plot of wt with H1 (Fig. 4b) shows that this clustering of *θ* is accompanied by a reduced protruding DNA arm contour length. This clearly indicates that histone H1 interaction results in further compaction of the DNA, as the changes in both parameters reveal an increased *l*_*w*_^40^. Therefore, Fig. 4c presents the distributions of calculated *l*_*w*_ around the histone core for wt nucleosomes with and without H1. The obtained mean of 145 bp ± 28 bp for *l*_*w*_ for wt without histone H1 is in agreement with comparable AFM studies^21,22,33,35,37^ and is a clear indication for the reliability of the here performed AFM experiments and the accuracy of QuantAFM also compared to the value of *l*_*w*_ determined by crystal structure analysis^3,4^. Histone H1 significantly increases the mean *l*_*w*_ ($p\ll 0.05$), shifting its distribution to a mean value of 163 bp ± 28 bp. This increase of DNA wrapping length of about 20 bp is in agreement with nuclease digestion experiments^12^ and molecular structures analysis^6,10^. From the observed changes of structural parameters extracted from the AFM images, we could confirm the binding of histone H1 to mono-nucleosomes and characterize the influence on the DNA entry/exit region resulting in a more compact conformation with increased DNA wrapping and therefore decreased DNA accessibility, which is in agreement with previous reports^6,7,60^.

Figure 4d shows the distributions of *l*_*w*_ for mutated nucleosomes with and without H1. The distribution of *l*_*w*_ of mutated nucleosome is, in comparison to wt, significantly shifted to lower values with a peak between 105 bp–120 bp ($p\ll 0.05$). The resulting mean value of 121 bp ± 32 bp supports earlier findings^24^, indicating a destabilizing effect of the H2A mutated nucleosomes. Histone H1 induced a significant increase in mean *l*_*w*_ ($p\ll 0.05$) of roughly 20 bp (143 bp ± 33 bp). The distribution of *l*_*w*_ of mut +H1 shows a clear peak between 120 bp–135 bp and more complexes with *l*_*w*_ above 150 bp compared to mut (38% of objects with H1 are above 150 bp, whereas only 16% of objects without H1). The increased number of complexes with DNA wrapping above 150 bp may represent a population of the mutated nucleosomes where H1 binding increases the compaction similarly to wt nucleosomes (68% of wt chromatosomes have wrapping above 150 bp). Further we see also a shift of roughly 15 bp in lower *l*_*w*_ regime. These changes confirm the influence of histone H1 for the mutated nucleosomes even after deposition on the PL coated surface. Our observations indicate that histone H1 induces compaction of the mutated nucleosomes, but the DNA accessibility in the chromatosomes is still substantially increased compared to wt. This suggests that histone H1 cannot completely compensate the altered nucleosome conformation induced by the charge-modifying mutations in H2A.

### Nucleosome positioning as indication for asymmetric unwrapping of mutated nucleosomes

Besides *θ* and *l*_*w*_, the positioning of nucleosomes can also be determined using QuantAFM. Previously it was shown that the positioning of the nucleosomes on the Widom-601 can be accurately determined in AFM experiments by analyzing the relative length of the protruding DNA arms^21,35^. In the present study, the positioning was characterized by determining the corrected short DNA arm ratio (sa-ratio_*c*_), which can be calculated from the automatically determined structural parameters by dividing the corrected contour length of the shorter protruding DNA arm by the sum of the corrected lengths of both protruding DNA arms (see AFM data evaluation). Therefore, the positioning of the nucleosome on the DNA is expressed via values ranging from 0 to 0.5 for the end to the central region of the DNA. For wt, the median of sa-ratio_*c*_ distribution was 0.468. This is close but somewhat smaller than the theoretical value, as we extended the Widom-601 with 153 bp and 164 bp overhangs resulting in a theoretical value of 0.483. The median of the sa-ratio_*c*_ for mutated nucleosomes is slightly lower, with a value of 0.455. In both cases, sa-ratio_*c*_ shows that the nucleosomes are located centrally on the DNA indicative for a strong positioning on the Widom-601 sequence. For comparing positioning, the small fraction of analyzed nucleosomes with sa-ratio_*c*_ < 0.3 was not considered (<1% for wt and 1% for mutated nucleosomes). This small fraction of nucleosomes positioned far apart from the Widom sequence is in agreement with Menshikova *et al*. They observed in an AFM study that a fraction of less than 5% of nucleosomes was positioned apart from the Widom-601^38^. The high affinity of the Widom-601 sequence was shown and utilized in several other AFM studies^21,22,35,40^.

However, we find a small but significant difference in the distributions of sa-ratio_*c*_ between wt and mutants (0.013 different median, $p\ll 0.05$). As described above, *l*_*w*_ is lower for mutated nucleosomes. An opening with symmetric increase in contour length of both protruding DNA arms would result in no change of the sa-ratio_*c*_. Therefore, the change in sa-ratio_*c*_ upon mutation can be explained by higher accessibility of one particular protruding DNA arm, indicating an asymmetric opening. As the Widom-601 sequence is non-palindromic, we named the two halves of the DNA *α* and *β*-side (Supplement Section C)^50^. For 464 bp DNA the data indicate that over the population of all mutated nucleosomes the by design longer *α*-side DNA arm is more accessible. This can be derived from the shift of the sa-ratio_*c*_ distribution to lower values compared to wt. However, as the difference between both DNA arms is only 11 bp (3.74 nm), which is inside the standard deviation of the measured contour lengths of the protruding DNA arms (Table 2), we cannot reliably distinguish which DNA arm belongs to which side (*α* or *β*). Therefore, we performed the same experiments with 599 bp long DNA fragments, to further investigate which DNA side becomes preferentially accessible upon mutation.

**Figure 5:**
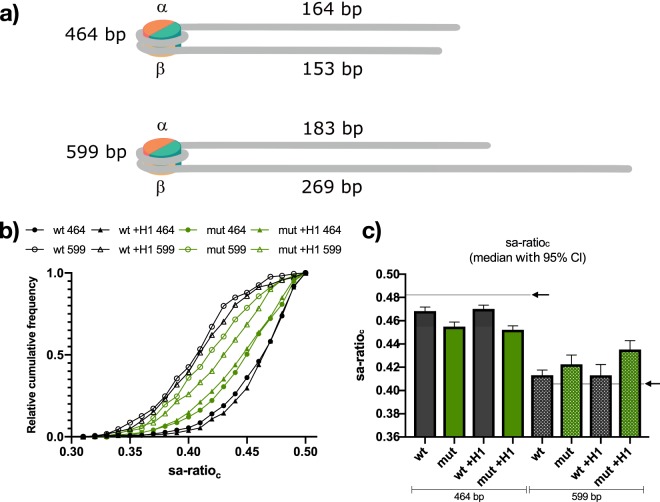
Nucleosome positioning. The positioning of the nucleosome objects on the DNA fragment is dominated by the Widom-601 positioning sequence. (**a**) The theoretical length of the DNA arms protruding from the octamer, consisting of a (H3–H4)_2_ tetramer (turquoise) and two H2A-H2B dimers (red and yellow) when positioned on the 147 bp long Widom- 601 sequence. The *α*-side corresponds for 464 bp nucleosomes to the longer and for 599 bp nucleosomes to the shorter DNA arm. This illustration displays the approximate length ratios between nucleosome core and protruding DNA arms. As nucleosome cores appear on the AFM images roughly twice the theoretical size (Table 2), ratios between the DNA arms were corrected (see Methods AFM data evaluation). (**b**) Cumulative frequency plot of the sa-ratio_*c*_ of all nucleosome samples reconstituted on 464 bp and 599 bp DNA. The cumulative frequency values of sa-ratio_*c*_, were normalized and binned (bin wide 0.01). (**c**) shows the median values with 95% confidence interval (CI) of the sa- ratio_*c*_ for the samples in (**b**). The theoretical values for positioning on the Widom-601 sequences are indicated by black arrows. For (**b** and **c**) only objects with a sa-ratio_*c*_ of at least 0.3 were considered: *n*_wt/464_ = 443, *n*_wt +H1/464_ = 443, *n*_mut/464_ = 439, *n*_mut +H1/464_ = 439, *n*_wt/599_ = 174, *n*_wt +H1/599_ = 185, *n*_mut/599_ = 183, *n*_mut +H1/599_ = 162. The shift between wt and mut for both 464 bp, and 599 bp DNA fragments indicate an asymmetric unwrapping of the *α*-side for the mutants.

We extended the Widom-601 sequence so that nucleosomes have two protruding DNA arms, which clearly differ in length (183 bp and 269 bp) resulting in a theoretical sa-ratio_*c*_ of 0.405. In contrast to the 464 bp fragments, for the 599 bp DNA the shorter DNA arm corresponds to the *α*-side (Fig. 5a). EMSA results as well as the trends of extracted structural parameters from automated AFM image analysis were the same for both (464 bp and 599 bp) DNAs (Supplementary Fig. S9). In Fig. 5b the calculated values of sa-ratio_*c*_ are plotted for all classes (464 bp and 599 bp DNA, with and without H1) as relative cumulative frequency curves. This representation of the sa-ratio_*c*_ distributions shows the differences in the positioning of wt and mutated nucleosome objects via the horizontal separation of the curves. The median of each distribution (sa-ratio_*c*_ value at 0.5 relative cumulative frequency) is plotted in Fig. 5c with highlighted values of theoretical sa-ratio_*c*_ (0.483 and 0.405, black arrows).

The shift to lower cumulative sa-ratio_*c*_ frequencies for wt samples reconstituted on 599 bp DNA (median of 0.413) in comparison to those reconstituted on 464 bp DNA (0.468) clearly demonstrates the positioning on the Widom-601 sequence. As mentioned, values for mutated 464 bp nucleosome objects are lower than those for wt, which is indicated in Fig. 5b by the separation of the curves. On the other hand, values of mutated nucleosome objects from 599 bp DNA (median of 0.423) are significantly shifted to higher values compared to wt ($p=0.007$). Histone H1 binding to nucleosomes (wt or mut) resulted in no significant shift of the sa-ratio_*c*_ distributions. But the evident difference in sa-ratio_*c*_ between wt and mutated samples is for both DNA fragments more distinct in the presence of H1, resulting in a difference of the median value of 0.018 and 0.022 for 464 bp and 599 bp samples, respectively. Although we have no direct marker for the corresponding DNA arm sides (*α* and *β*) the experimental setup with the two differently designed DNA fragments strongly indicates that for mutants with and without H1 one side (the *α*-side) is preferentially more accessible. This implies that H2A mutated nucleosomes are more susceptible to DNA sequence dependent opening, even in the presence of H1.

Our results reveal that the enhanced DNA accessibility upon mutation with and without histone H1, indicated by increased DNA opening angle and decreased DNA wrapping length cohere with an asymmetric DNA unwrapping on the less bendable *α*-side. This asymmetric unwrapping from the *α*-side was reported earlier by other techniques^48–51^. In the present study, using our automated image analysis method, we found this phenomenon via the comparison of AFM images from wt and H2A mutated nucleosome complexes absorbed to PL coated surface. Thus, we suggest that this mutation enhances the effect of intrinsic asymmetry of the Widom-601 sequence.

## Discussion

Gene regulation is highly influenced by DNA compaction via histone proteins. A growing number of *in vivo* and *in vitro* studies describe the impact of factors like the DNA sequence on the dynamics of nucleosomes as well as the role of nucleosome dynamics on the accessibility of DNA^61^. For *in vitro* studies, the most often used nucleosome positioning sequence is the Widom-601^25^. Recently, the intrinsic asymmetry of the Widom-601 resulting in a preferred DNA unwrapping of the less bendable *α*-side was found using optical tweezers on nucleosomes attached to the surface^51^. Subsequently, we could show the same effect for freely diffusing nucleosomes in NaCl induced destabilization assays^50^. Monte Carlo simulations supported by SAXS and FRET measurements has revealed the same sequence dependent asymmetric opening for core particles on the Widom-601 but not on the more random 5S DNA^48^. In the present study, via accurate analysis of AFM images of PL absorbed wt and mutated nucleosome complexes using our automated image analysis method QuantAFM, we found that DNA accessibility is asymmetrically enhanced upon this mutation. In comparison to wt, mutated nucleosomes with and without histone H1 show a more open conformation, where preferentially the *α*-side has opened. We conclude this from three characteristics deduced from automatically extracted structural parameters of more than 2000 analyzed individual nucleosome objects: 1) In contrast to wt, mutated nucleosomes with and without H1 show a rather open “beads on a string” conformation with opening angles above 90° (Fig. 3). 2) For mutated nucleosomes without and with histone H1 *l*_*w*_ was clearly decreased in comparison to wt (Fig. 4). 3) Our analysis of the nucleosome positioning reveals that especially the contour length of the *α*-side DNA arm was increased in mutated nucleosome complexes compared to wt (Fig. 5). Similar comparisons of the positioning of wt nucleosomes and nucleosomes containing different H2A variants reconstituted on the Widom-601 were done in several AFM studies^21,22,62^. DNA wrapping was also reduced compared to wt for nucleosomes containing H2A.Bdb variant^21,22^. Interestingly, the relative positioning on the DNA fragments did not change and therefore a symmetric increase in DNA arm length was suggested. Although time-lapse and high-speed AFM are suitable to monitor the nucleosome unwrapping process^37,40,63,64^, this dominant *α*-side unwrapping of mono-nucleosomes reconstituted on the Widom-601 was to our knowledge not yet reported for AFM. This underlines that the conformation with preferentially opened *α*-side is a feature of our mutated nucleosomes on the Widom-601, which is preserved upon surface immobilization and drying applied for AFM imaging.

Earlier we suggested a pathway of nucleosome unwrapping from the Widom-601 sequence, which starts with the linker DNA opening on the *α*-side, followed by eviction of the H2A-H2B dimer on this opened side^50^. Results for nucleosomes with the milder mutation (H2A R81A) revealed that the suggested unwrapping pathway is accelerated upon mutation in the *α*3 helices of histone H2A^50^. Our study supports the findings obtained by single molecule FRET^50^, via visualization of the dominantly more accessible *α*-side of H2A R81E/R88E mutants using AFM. In this context, a recent cryo-EM study of partially unwrapped nucleosome states gave interesting structural insights on how the histone octamer adapts to opening DNA arm via plasticity and reorientation^65^. This study could show in agreement with^50^ that the H2A-H2B dimer on the opened DNA side shows more dynamics in contrast to the dimer at the wrapped DNA side. Moreover, it was evident that the reorientation of the H2A-H2B dimer is important for interacting and stabilization of partially unwrapped DNA arm^65^. This suggests that due to the destabilized dimer/tetramer interface and changed electrostatic environment for H2A R81E/R88E nucleosomes^24,66^, the interaction to the less bendable, opening DNA arm (*α*-side), is altered, which results in the observed open conformation. Similar to our findings, Doyen *et al*. described in EM experiments an open conformation for nucleosomes containing H2A.Bbd variant, which showed also a reduced DNA wrapping in AFM experiments^20^. Shukla *et al*. expanded these studies on the H2A.Bbd variant investigating the interaction with histone H1. They suggested an impeded binding of histone H1 to H2A.Bdb containing nucleosomes^22^. In contrast to the H2A.Bbd nucleosomes, histone H1 binding could be confirmed for H2A R81E/R88E mutated nucleosomes via extraction and comparison of structural features collected by AFM and also by EMSA. Further, our experiments with fluorescently labeled histone H1 verified the presence of linker histone after deposition for AFM experiments (Supplementary Fig. S7). However, the portion of mutated chromatosomes which showed *l*_*w*_ above 150 bp (38%) was significantly decreased compared to wt chromatosomes (68%). Bednar *et al*. found on the basis of the structural information of a 197 bp chromatosome that linker DNA arm flexibility can influence histone H1 interaction. They suggested that factors that affect DNA arm entry/exit angle could have an impact on histone H1 interaction as they observed a loss of interaction to one of the linker DNA^6^. In this context, it is a striking observation that the difference between wt and mutants in apparent positioning was more distinct in the presence of H1. We hypothesize that the interaction between histone H1 and the DNA arm is disrupted whereas the *β*-side is unaffected, leading to the pronounced difference in positioning. Therefore, our findings unveil that the impact of the influence of DNA sequence properties on nucleosome dynamics can be amplified by internal nucleosome modification or vice versa, which could not completely be compensated by histone H1 interaction. However, the determined values for wt chromatosomes underline the histone H1 induced compaction. This is to our knowledge the first report in AFM studies describing the impact of histone H1 on DNA wrapping and DNA opening angle in a consistent manner. With the automated detection and feature extraction of nucleosome-like objects, our here presented image analysis method QuantAFM demonstrated its ability to facilitate quantification of AFM images and could be adapted and improved with regard to similar investigation.

## Methods

### DNA fragments

The 464 bp and 599 bp DNA fragments containing the 147 bp long Widom-601 sequence^25^ were produced via PCR on the pGEM-3z/601 plasmid (Widom-601, Addgene plasmid #26656; http://n2t.net/addgene:26656; RRID:Addgene_26656) and purified on Gen-Pak FAX HPLC (Waters). The primers (Sigma Aldrich, USA) as well as the DNA sequence of the two fragments are listed in (Supplementary Section C).

### Histone proteins

The recombinant *Xenopus laevis* core histones were purchased from Planet Protein (Colorado State University, USA). Mutated *Xenopus laevis* histone H2A (R81E/R88E) as well as recombinant *Human* linker histones (H1.0a) were expressed in *E*. *coli*, purified, and the histone H1-T77C was labeled with Alexa488-maleimide (H1-T77C-Alexa488) as described^24^.

### Nucleosome experiments

The histone octamers were reconstituted following the protocols described earlier^24,67^. Nucleosomes with and without histone H1 were reconstituted in a molar ratio of DNA: octamer of 1:2 for the 464 bp DNA and 1:3 for 599 bp DNA via salt dialysis as described earlier^24,67^. Briefly, DNA and octamers were mixed in (10 mM Tris-HCl, 0.1 mM EDTA, 2 M NaCl) and slowly dialyzed at 4 °C in Slide-A Lyzer Mini Dialysis Units, 7000 MWCO (Thermo Scientific, USA) down to 5 mM NaCl concentration. For reconstitution of chromatosomes, histone H1 was added in excess up to a molar ratio of 1:4 (DNA: histone H1) during the reconstitution process at NaCl concentration around 600 mM. Reconstitution was controlled via agarose gel electrophoresis. Samples were loaded together with 1 kb DNA ladder (Bioron, Germany) on 1% agarose gels in TBE buffer (pH 7.5). After electrophoresis, gels were incubated for 15 min in an ethidium bromide solution and imaged under UV light.

### AFM experiments

AFM experiments in air were performed after established protocols using Poly(L)lysine (PL) (Sigma-Aldrich) modified mica surfaces^58^. Therefore, 30 *μ*l of a 10 *μ*g/*μ*l PL solution was deposited for 1–2 min on the freshly cleaved mica (Plano, Germany). After incubation, the surface was washed with 1.5 ml ddH_2_O and dried under a nitrogen stream. Afterwards, 30 *μ*l of the nucleosome suspension (10 mM Tris- HCl, 0.1 mM EDTA, 15 mM NaCl, pH 7.5 and 1 nM–5 nM nucleosome concentration) was deposited on the surface and immediately washed with 1.5 ml ddH_2_O and dried under nitrogen stream. Measurements were performed using Nanoscope V, Digital Instruments (Santa Barbara, USA) with Nanoscope software (version 7.13, Veeco Instruments) running in tapping mode with silicon probes (type: PointProbePlus, NCH, Nanoworld, Switzerland) at drive frequencies of 300 kHz–400 kHz with a set point between 2.0 V–3.0 V. The scan rate was 1.36 Hz with 2 *μ*m × 2 *μ*m scan size and 1024 × 1024 image size. AFM images were flattened and converted into 8-bit tagged image file format (TIFF). Experiments with different DNA sizes were performed in at least three replicates each (replicates are nucleosome reconstitutions, controlled via EMSA and AFM imaging with at least two independent fixations on the PL coated mica surface).

### AFM data evaluation

For automated analysis of AFM data, our image analysis method QuantAFM (see Supplementary Section A) with standard thresholding and background threshold level of 90 was used. The range of radii for Hough voting was set to 8.5 nm to 14.0625 nm, and the sensitivity for Hough voting was varied between 0.90 and 0.96. To determine the opening angle *θ*, line fitting for 5 pixels (roughly 10 nm) was performed. For contour length determination, the average length was computed. For the other parameters default values were employed. For comparative analysis of nucleosome objects, the same parameter setting was used for all data. Only objects within the expected contour length range (464 bp, 65 nm–155 nm; 599 bp, 130 nm–190 nm) which showed no major abnormalities (wrong DNA backbone path, intersections, strong contaminations) were included in the analysis, the other objects were discarded. From every automatically analyzed image, quantified parameters of all valid objects, as well as manually determined parameters for objects, where the automated image analysis method could not determine all parameters (below 25% of all evaluated objects), were used for our evaluation. Supplementation with manual analysis was done to ensure that all object conformations on the images are considered. Therefore, we used at least 60% of the valid objects on the analyzed images for our evaluation. In cases where QuantAFM yielded a wrong opening angle *θ* (identified by visual inspection), *θ* was replaced by the inverse angle 180 − *θ*. Manual image analysis was performed using the Fiji software^68^. Contour lengths were determined by tracing centrally through the fragments using the Segmented Line Selection Tool, and *θ* was determined using the Angle tool. The nm/bp ratio, which is the distance in nm between two bp of the DNA, was determined by measuring the contour length of free DNA fragments. The mean value of the contour length of DNA filaments was divided by the number of bp of the corresponding DNA fragment. Due to the broadening artifacts in AFM measurements, the radius of a nucleosome appears to be approximately twice as large as its known radius (Table 2). Hence, for an estimation of the DNA wrapping length and nucleosome positioning, the length of each protruding DNA arm needs to be corrected^33^ by adding the difference between the automatically determined radius of an individual object and the known radius of the nucleosome (5.5 nm)^3^. The DNA wrapping length *l*_*w*_ was calculated by subtracting the corrected contour length of the protruding DNA arms from the mean value of the free DNA fragment length and then converted into bp via division with the nm/bp ratio (0.34 nm/bp for our experiments). The positioning of the nucleosomes on the DNA fragments was quantified by the corrected short DNA arm ratio (sa-ratio_*c*_), which is a slightly modified version of the r-ratio described earlier^33^. For each individual object, the corrected length of the short protruding DNA arm was divided by the sum of both corrected protruding DNA arms, resulting in a sa-ratio_*c*_ ranging from 0 to 0.5.

### Statistical data analysis

To analyze and compare the parameters of the DNA and nucleosome classes the mean value (mean) with standard deviation (sd) or median with 95% confidential interval (CI) were calculated. For statistical analysis, distributions were tested for normality using Shapiro-Wilk normality test. Normal distributed data were compared using unpaired two-tailed *t*-test, otherwise Mann-Whitney U test was used. Multiple comparisons were done using One-way ANOVA or Kruskal-Wallis test with following Tukey’s post-test or Dunn’s post test, respectively. The significance level was $p\, < \,0.05$. The *p*-values for the comparison of DNA opening angles, DNA wrapping length and sa-ratio_*c*_ were determined using Kruskal-Wallis test with subsequent Dunn’s post test. Data were analyzed using GraphPad Prism version 8.1.2 for Mac (GraphPad Software, La Jolla California USA).

### Test experiments with fluorescently labeled histone H1

Persistence of histone H1 binding to nucleosomes after absorption to PL coated surface (for AFM imaging protocol) was evaluated with fluorescently labeled histone (H1-T77C-Alexa488). The fluorescence signal of the samples on PL coated circular glass slides (8 mm diameter coverslips) was detected with Typhoon multimode imager (GE Healthcare, Germany). The excitation was at 488 nm wavelength with BP 520/40 nm emission filter. The fluorescence intensity was measured by setting the amplification to 650 V and the image was scanned with 100 *μ*m resolution (see Supplementary Section D).

## Supplementary information

Supplementary Information

## Data Availability

The source code of our software as well as example images are available online (https://github.com/dennlinger/quantAFM). The whole datasets generated and analyzed during the current study are available from the corresponding author on reasonable request.

## References

[1] Olins AL, Olins DE (1974). Spheroid chromatin units (v bodies). Science (New York, NY).

[2] Van Holde KE (1989). Chromatin.

[3] Luger K, Mader AW, Richmond RK, Sargent DF, Richmond TJ (1997). Crystal structure of the nucleosome core particle at 2.8 A resolution. Nature.

[4] Vasudevan D, Chua EY, Davey CA (2010). Crystal structures of nucleosome core particles containing the ‘601’ strong positioning sequence. J Mol Biol.

[5] Kinkade, J. & Cole, R. The resolution of four lysine-rich histones derived from calf thymus. *J Biol Chem* **241** (1966). 5954358

[6] Bednar J (2017). Structure and dynamics of a 197 base-pair nucleosome in complex with linker histone H1. Molecular Cell.

[7] Hamiche A, Schultz P, Ramakrishnan V, Oudet P, Prunell A (1996). Linker Histone-dependent DNA Structure in Linear Mononucleosomes. Journal of Molecular Biology.

[8] Parseghian MH (2015). What is the role of histone H1 heterogeneity? A functional model emerges from a 50 year mystery. AIMS Biophysics.

[9] Syed SH (2010). Single-base resolution mapping of H1-nucleosome interactions and 3D organization of the nucleosome. Proceedings of the National Academy of Sciences of the United States of America.

[10] Zhou B-R (2013). Structural insights into the histone H1-nucleosome complex. Proceedings of the National Academy of Sciences.

[11] Zhou YB, Gerchman SE, Ramakrishnan V, Travers A, Muyldermans S (1998). Position and orientation of the globular domain of linker histone H5 on the nucleosome. Nature.

[12] Simpson RT (1978). Structure of the chromatosome, a chromatin particle containing 160 base pairs of DNA and all the histones. Biochemistry.

[13] Cutter AR, Hayes JJ (2015). A brief review of nucleosome structure. FEBS letters.

[14] Rippe, K., Mazurkiewicz, J. & Kepper, N. *Interactions of Histones with DNA: Nucleosome Assembly*, *Stability*, *Dynamics*, *and Higher Order Structure*, chap. 6, 135–172 (John Wiley & Sons, Ltd, 2007).

[15] Bannister AJ, Kouzarides T (2011). Regulation of chromatin by histone modifications. Cell Research.

[16] Bonisch C, Hake SB (2012). Histone H2A variants in nucleosomes and chromatin: more or less stable?. Nucleic Acids Res.

[17] Maze I, Noh K-M, Soshnev AA, Allis CD (2014). Every amino acid matters: essential contributions of histone variants to mammalian development and disease. Nat Rev Genet.

[18] Montel F (2009). The Dynamics of Individual Nucleosomes Controls the Chromatin Condensation Pathway: Direct Atomic Force Microscopy Visualization of Variant Chromatin. Biophysical Journal.

[19] Chadwick BP, Willard HF (2001). A novel chromatin protein, distantly related to histone H2A, is largely excluded from the inactive X chromosome. The Journal of Cell Biology.

[20] Doyen C-M (2006). Dissection of the unusual structural and functional properties of the variant H2A.Bbd nucleosome. The EMBO Journal.

[21] Montel F, Fontaine E, St-Jean P, Castelnovo M, Faivre-Moskalenko C (2007). Atomic force microscopy imaging of SWI/SNF action: mapping the nucleosome remodeling and sliding. Biophys J.

[22] Shukla MS (2011). The docking domain of histone H2A is required for H1 binding and RSC-mediated nucleosome remodeling. Nucleic Acids Res.

[23] White AE, Hieb AR, Luger K (2016). A quantitative investigation of linker histone interactions with nucleosomes and chromatin. Scientific Reports.

[24] Lehmann K (2017). Effects of charge-modifying mutations in histone H2A alpha3-domain on nucleosome stability assessed by single-pair FRET and MD simulations. Scientific Reports.

[25] Lowary PT, Widom J (1998). New DNA sequence rules for high affinity binding to histone octamer and sequence-directed nucleosome positioning. J Mol Biol.

[26] Binnig G, Rohrer H (1983). Scanning tunneling microscopy. Surface Science.

[27] Hansma HG, Bezanilla M, Zenhausern F, Adrian M, Sinsheimer RL (1993). Atomic force microscopy of DNA in aqueous solutions. Nucleic Acids Research.

[28] Ikai A (1996). STM and AFM of bio/organic molecules and structures. Surface Science Reports.

[29] Lyubchenko Yuri L., Shlyakhtenko Luda S., Gall Alexander A. (2009). Atomic Force Microscopy Imaging and Probing of DNA, Proteins, and Protein-DNA Complexes: Silatrane Surface Chemistry. Methods in Molecular Biology™.

[30] Lyubchenko YL, Shlyakhtenko LS (2016). Imaging of DNA and Protein–DNA Complexes with Atomic Force Microscopy. Critical Reviews in Eukaryotic Gene Expression.

[31] Bussiek M, Toth K, Schwarz N, Langowski J (2006). Trinucleosome compaction studied by fluorescence energy transfer and scanning force microscopy. Biochemistry.

[32] Kizaki S, Suzuki Y, Takenaka T, Endo M, Sugiyama H (2014). AFM analysis of changes in nucleosome wrapping induced by DNA epigenetic modification. Biomaterials Science.

[33] Kepert JF (2003). Conformation of reconstituted mononucleosomes and effect of linker histone H1 binding studied by scanning force microscopy. Biophys J.

[34] Zou T (2018). Direct Observation of H3–H4 Octasome by High-Speed AFM. Chemistry - A European Journal.

[35] Nazarov I (2016). AFM studies in diverse ionic environments of nucleosomes reconstituted on the 601 positioning sequence. Biochimie.

[36] Rychkov GN (2017). Partially Assembled Nucleosome Structures at Atomic Detail. Biophysical Journal.

[37] Stumme-Diers MP, Banerjee S, Hashemi M, Sun Z, Lyubchenko YL (2018). Nanoscale dynamics of centromere nucleosomes and the critical roles of CENP-A. Nucleic Acids Research.

[38] Menshikova I, Menshikov E, Filenko N, Lyubchenko YL (2011). Nucleosomes structure and dynamics: effect of CHAPS. International Journal of Biochemistry and Molecular Biology.

[39] Lyubchenko YL (2014). Nanoscale Nucleosome Dynamics Assessed with Time-lapse AFM. Biophys Rev.

[40] Shlyakhtenko LS, Lushnikov AY, Lyubchenko YL (2009). Dynamics of nucleosomes revealed by time-lapse atomic force microscopy. Biochemistry.

[41] Fang Y (1998). Solid-State DNA Sizing by Atomic Force Microscopy. Analytical Chemistry.

[42] Ficarra E, Benini L, Macii E, Zuccheri G (2005). Automated DNA fragments recognition and sizing through AFM image processing. IEEE Trans Inf Technol Biomed.

[43] Ficarra E (2005). Automatic intrinsic DNA curvature computation from AFM images. IEEE Trans Biomed Eng.

[44] Rivetti C, Codeluppi S (2001). Accurate length determination of DNA molecules visualized by atomic force microscopy: evidence for a partial B- to A-form transition on mica. Ultramicroscopy.

[45] Sanchez-Sevilla A, Thimonier J, Marilley M, Rocca-Serra J, Barbet J (2002). Accuracy of AFM measurements of the contour length of DNA fragments adsorbed on mica in air and in aqueous buffer. Ultramicroscopy.

[46] Spisz, T. S. *et al*. Automated sizing of DNA fragments in atomic force microscope images. *Med Biol Eng Comput* (1998). 10.1007/BF02518867 10367455

[47] Sundstrom A (2012). Image Analysis and Length Estimation of Biomolecules Using AFM. IEEE Transactions on Information Technology in Biomedicine.

[48] Mauney AW, Tokuda JM, Gloss LM, Gonzalez O, Pollack L (2018). Local DNA Sequence Controls Asymmetry of DNA Unwrapping from Nucleosome Core Particles. Biophysical journal.

[49] Chen Y (2017). Asymmetric unwrapping of nucleosomal DNA propagates asymmetric opening and dissociation of the histone core. Proceedings of the National Academy of Sciences.

[50] Gansen A (2018). High precision FRET studies reveal reversible transitions in nucleosomes between microseconds and minutes. Nature Communications.

[51] Ngo TTM, Zhang Q, Zhou R, Yodh JG, Ha T (2015). Asymmetric unwrapping of nucleosomes under tension directed by DNA local flexibility. Cell.

[52] Marek J (2005). Interactive measurement and characterization of dna molecules by analysis of afm images. Cytometry Part A.

[53] Buades A, Coll B, Morel J-M (2011). Non-Local Means Denoising. Image Processing On Line.

[54] Otsu N (1979). A threshold selection method from gray-level histograms. IEEE Transactions on Systems, Man, and Cybernetics.

[55] Lam L, Lee S-W, Suen CY (1992). Thinning methodologies-a comprehensive survey. IEEE Trans. Pattern Anal. Mach. Intell..

[56] Kulpa Z (1977). Area and perimeter measurement of blobs in discrete binary pictures. Computer Graphics and Image Processing.

[57] Duda RO, Hart PE (1972). Use of the Hough Transformation to Detect Lines and Curves in Pictures. Commun. ACM.

[58] Bussiek M, Mucke N, Langowski J (2003). Polylysine-coated mica can be used to observe systematic changes in the supercoiled DNA conformation by scanning force microscopy in solution. Nucleic Acids Res.

[59] Bussiek M, Muller G, Waldeck W, Diekmann S, Langowski J (2007). Organisation of nucleosomal arrays reconstituted with repetitive African green monkey alpha-satellite DNA as analysed by atomic force microscopy. Eur Biophys J.

[60] Fan L, Roberts VA (2006). Complex of linker histone H5 with the nucleosome and its implications for chromatin packing. Proceedings of the National Academy of Sciences of the United States of America.

[61] Lai WKM, Pugh BF (2017). Understanding nucleosome dynamics and their links to gene expression and DNA replication. Nature reviews. Molecular Cell Biology.

[62] Syed SH (2009). The incorporation of the novel histone variant H2AL2 confers unusual structural and functional properties of the nucleosome. Nucleic Acids Research.

[63] Miyagi A, Ando T, Lyubchenko YL (2011). Dynamics of nucleosomes assessed with time-lapse high-speed atomic force microscopy. Biochemistry.

[64] Lyubchenko YL, Shlyakhtenko LS (2009). AFM for analysis of structure and dynamics of DNA and protein-DNA complexes. Methods (San Diego, Calif.).

[65] Bilokapic S, Strauss M, Halic M (2018). Histone octamer rearranges to adapt to DNA unwrapping. Nature Structural & Molecular Biology.

[66] Biswas M, Voltz K, Smith JC, Langowski J (2011). Role of histone tails in structural stability of the nucleosome. PLoS Comput Biol.

[67] Böhm V (2011). Nucleosome accessibility governed by the dimer/tetramer interface. Nucleic Acids Res.

[68] Schindelin J (2012). Fiji: an open-source platform for biological-image analysis. Nature Methods.

