# Supplementary material for: DNA accessibility of chromatosomes quantified by automated image analysis of AFM data
Source: Sci Rep. 2019 Sep 4;9:12788. doi: 10.1038/s41598-019-49163-4 (PMC6726762; doi:10.1038/s41598-019-49163-4)
Supplement: Supplementary file 1 — Supplementary Information [file 41598_2019_49163_MOESM1_ESM.pdf]

Supplementary Material: DNA accessibility of chromatosomes quantified by automated image analysis of AFM data

Martin Würtz[1,3], Dennis Aumiller[2], Lina Gundelwein[2], Philipp Jung[2], Christian Schütz[2], Kathrin Lehmann[1,5], Katalin Tóth[1,+], and Karl Rohr[3,4,+,*]

[1]German Cancer Research Center, Division Biophysics of Macromolecules, Heidelberg, 69120, Germany
[2]Heidelberg University, Institute of Computer Science, Heidelberg, 69120, Germany
[3]Heidelberg University, BioQuant and IPMB, Biomedical Computer Vision Group, Heidelberg, 69120, Germany
[4]German Cancer Research Center, Heidelberg, 69120, Germany
[5]Simon Fraser University, Department of Physics, Burnaby, BC, V5A 1S6, Canada
[*]k.rohr@dkfz.de
[+]these authors contributed equally to this work

We provide algorithmic details of the developed image analysis method, a description of the performance evaluation, as well as supporting information regarding AFM experiments.

A Algorithm details of the image analysis method QuantAFM

In this section, we provide algorithmic details of the developed image analysis method for automated analysis and quantification of AFM data. The method is implemented in MATLAB. For the Non-Local Means denoising step OpenCV is used. The implemented software can be found in our repository at https://github.com/dennlinger/quantAFM.

A.1 Denoising and filtering

Preprocessing of the original images is performed using Non-Local Means Denoising[1] from OpenCV. This removes a significant part of background noise before applying standard filtering. The Non-Local Means approach proved to be more effective than other denoising algorithms, such as wavelet decomposition and Wiener filtering. In particular, the non-locality and intensity similarity measure of the approach is crucial to maintain the structure and contrast of the filaments to ensure correct subsequent segmentation by thresholding. In comparison, Sundstrom *et al.* compare the respective pixel intensities to a local mean during the thresholding step[2]. In our method, by leveling the background before the thresholding step, the distinction between background and filaments becomes much clearer, due to increasing contrast as can be seen in Fig. S1.

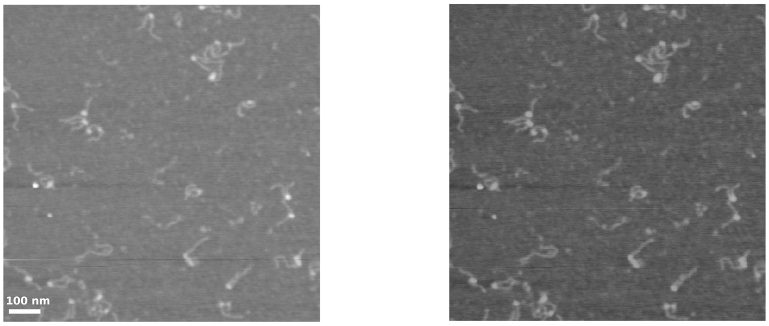

(a) (b)

Figure S1. Example image section (a) before and (b) after denoising using Non-Local Means. Note the overall gain in contrast.

After Non-Local Means Denoising, a 3×3 median filter is applied to remove remaining noise. Furthermore, we implemented an adaptive low pass filter using the Fourier transform, which operates on the amplitude representation in frequency space of the whole image. By selecting only lower frequencies, the image is generally better suited for segmentation. An advantage of the smoothing effect of the filter is that thin filaments have more similar intensities than before. On the other hand, the overall image intensity is lowered due to the high background/foreground intensity ratio. In some cases with very thin filaments or very low overall intensity values this might render segmentation difficult. An advantage of using the Fourier transform is that the horizontal movement of the AFM tip is highlighted in the frequency domain and can thus be mostly eliminated. In the filtered image, the horizontal artifacts are reduced leading to an improved connectivity of the objects in the image. Also, compared to convolution with a fixed size filter in space domain, the low pass filter in the frequency domain affects the whole image and only needs to be computed once for a single image.

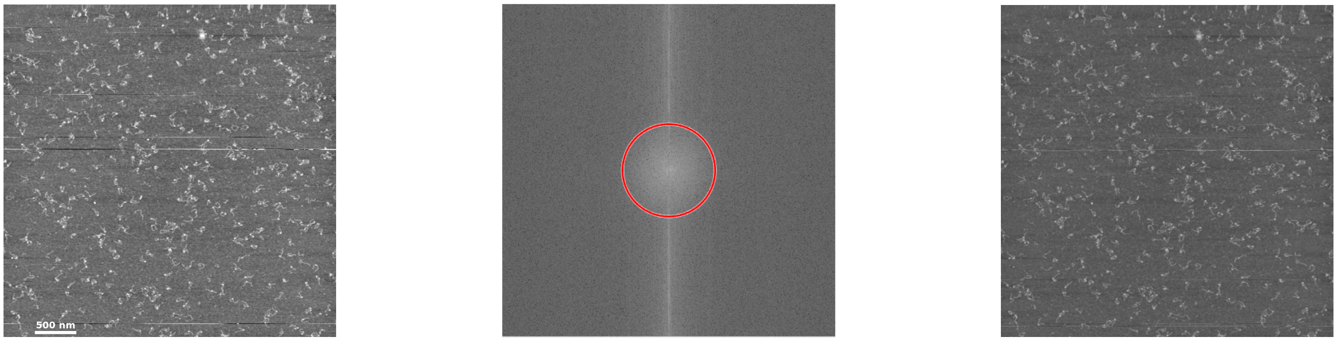

(a) (b) (c)

Figure S2. (a) Original image, (b) amplitude representation in frequency space with low pass filter region (inside red circle), and (c) image after median and low pass filtering.

A.2 Nucleosome detection

Nucleosome structures are visible as circular structures attached to filament strands. Hence, we use the Hough Transform[3] with prior edge detection on the gray-value images to detect circles within a range of radii specified by the user. This yields position and radii of the detected circles. The range of radii is chosen according to the properties of the analyzed filaments and should be limited as much as possible to improve the runtime and accuracy.

A.3 Thresholding

We tested a wide variety of thresholding algorithms using a number of different implementations in GNU Octave[1]. Generally, the best result was obtained by Otsu's method[4]. Otsu's method determines the global threshold of an image by minimizing the intensity variance within the background class and foreground class. Using Otsu's method, we developed an iterative thresholding method that proved to perform well for the considered AFM images. Since the number of high quality images in AFM image sets is generally rather limited, we wanted to ensure that low quality images can be processed as well. To cope with a large variety of noise and artifacts, our iterative thresholding method performs the following steps after denoising:

1. The background intensity is leveled out by setting all intensity values below a global average value to the same background value.
2. An initial binarization step with a threshold determined by Otsu's method is performed to identify outliers in terms of object size. These outliers are removed from the image by setting the respective region to the background intensity.
3. Afterwards, the average threshold is computed globally for the whole dataset and intensity values outside the range of 1.5σ (standard deviation) above or below the threshold are discarded. This step accounts for changes in the overall average intensity.

We note that the last step performs best for data sets acquired under similar conditions and a relevant number of images. After performing iterative thresholding, a second binarization is performed with the updated threshold. Steps 1 and 2 of our iterative thresholding method removes outliers compared to Otsu's method. For some data, particular images with strong contamination (see Fig. S3(a)), the result still needs to be improved, which is why we further restrict the threshold range to

[1] The specific ones where: *intermetodes, percentile, minimum, MaxEntropy, MaxLikelihood, intermeans, moments, minerror, concavity, Otsu*

1.5σ in step 3. Our iterative thresholding method leads to clear improvements compared to Otsu's method for strong noise and overall noisy backgrounds (see Fig. S3(b) and (d)). Using instead of 1.5σ a different threshold range yields worse results (see Fig. S3(c) for 1σ). Additionally, we provide the option to threshold single images in case the globally determined threshold for the whole data set does not result in satisfying segmentations.

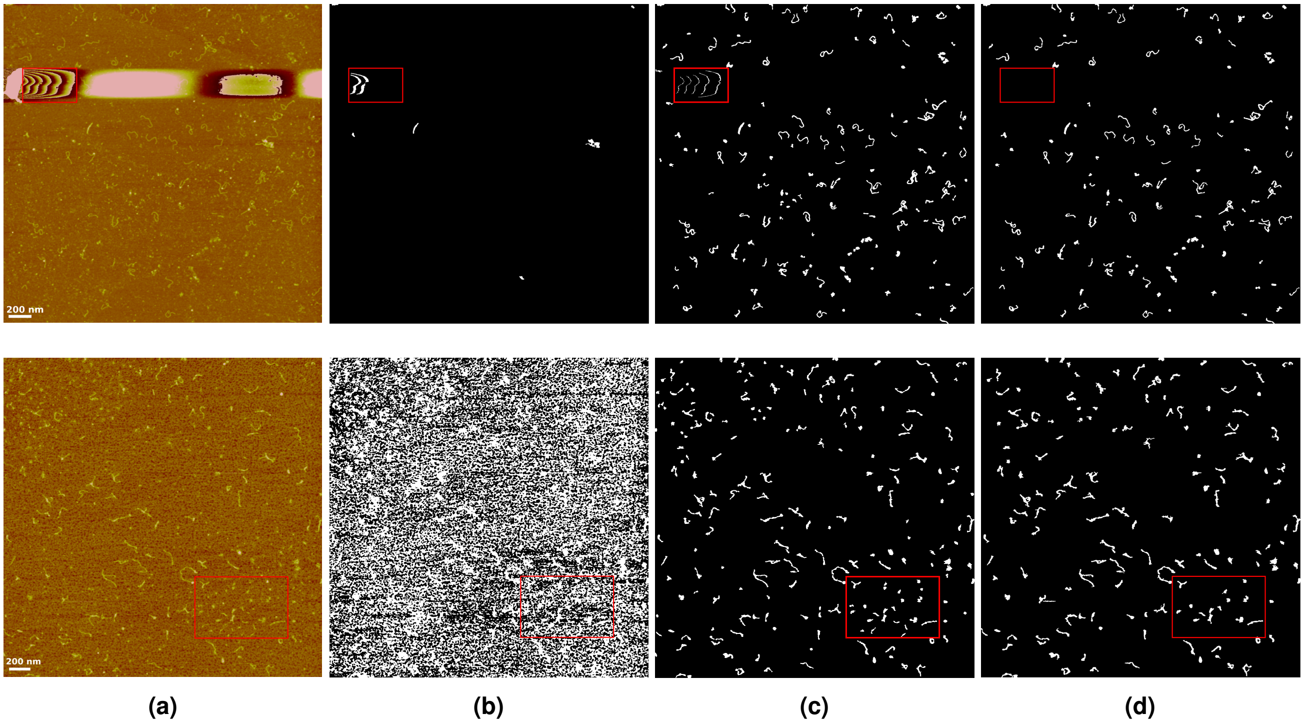

Figure S3. Thresholding results for two example images (top, bottom). (a) Original image, (b) result of standard Otsu's method, (c) resulting binary image using our iterative thresholding method with a threshold of 1σ, and (d) results for iterative thresholding with 1.5σ. Red boxes highlight regions with clear differences and artifacts.

A.4 Thinning

The skeletons of the filament structures are extracted by Hilditch's sequential thinning algorithm[5], which provided slightly better results than an implementation of Zhang and Suen's parallelized thinning algorithm[6]. The resulting skeletons were generally smoother compared to Zhang-Suen's algorithm which in some cases yields skeletons with a width of more than one pixel. Hilditch's algorithm yielded reliably one pixel-width strands (see Fig. S5), which is a necessary requirement for the following length estimations.

A.5 Graph-based length determination

By representing a thinned filament strand as a graph, where the vertices correspond to the image pixels and edges are the adjacency relations of these vertices (i.e., neighboring pixels are connected), the length of a filament can be determined by double Single Source Shortest Path (SSSP)[2]. More specifically, via Breadth-First Search (BFS) by starting from an arbitrary vertex in the graph and calculating the furthest point, we end up at either one of the true endpoints of the filament. This only holds under the assumption that there are no circular substructures within the thinned object, hence such cases are discarded as invalid beforehand. The second BFS iteration of SSSP will lead from the determined endpoint to the other end of the strand, since this is per definition the furthest point from here, as shown in Fig. S4.

In our method the computed length is corrected by the Kulpa estimator to account for a discrepancy in the discrete representation[7,8]. Additionally, re-computing the endpoints based on the orientation of the outermost pixels of the thinned strand is also possible. Since the thinning also removes parts of the ends of the filament, the length is otherwise slightly underestimated, as can be seen for the lower left arm of Fig. S6. Elongation of the filaments is performed until the border of the thick filament is reached. We obtained the best performance by calculating the mean value of both the computed strand, and its re-computed equivalent ("average mode"), which we included as the default parameter setting. In the case of nucleosome-like objects our method computes the total contour length as well as the contour length of each protruding DNA arm (measured from the intersection of each DNA arm with the detected nucleosome circle).

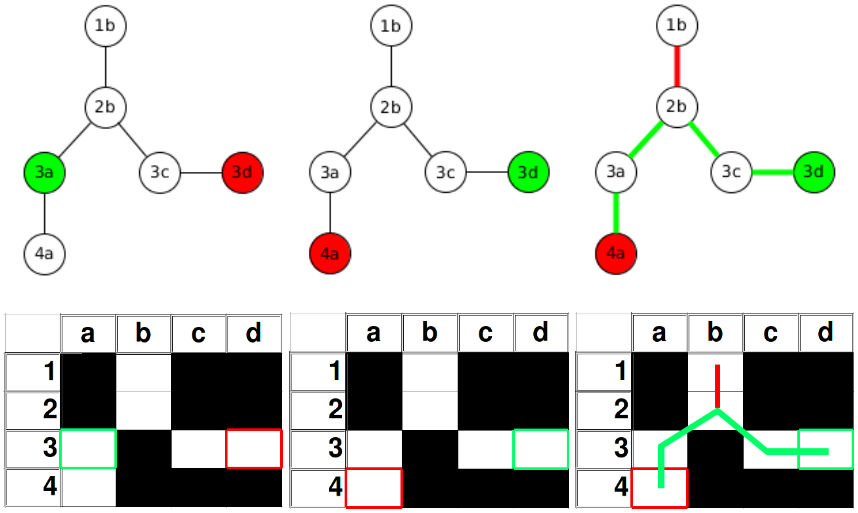

(a) First BFS **(b)** Second BFS **(c)** Shortest path

Figure S4. Example of the graph-based length determination as graphical representation (top row) and as pixel map (bottom row). (a) Randomly chosen starting node (green) and endpoint determined by SSSP (red), (b) second iteration of SSSP with starting point (green) determined in previous iteration and successively found new endpoint (red), (c) final path between starting and endpoint (green line) and rejected part of the filament (red line).

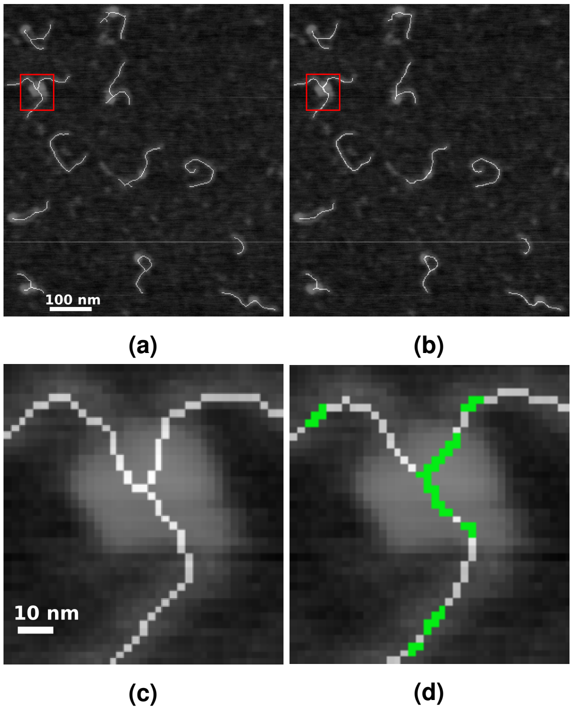

Figure S5. Overlay of extracted skeletons using (a) Hilditch's algorithm and (b) Zhang-Suen's algorithm. Respective enlarged regions marked in top row are shown for (c) Hilditch's algorithm and (d) Zhang-Suen's algorithm, where the latter yields skeletons with a width of more than one pixel (highlighted in green).

A.6 Angle determination

The angle determination is performed only if a single nucleosome is attached to a DNA filament. Otherwise, only the total contour length will be determined. The first step is to determine the intersection of the nucleosome with the filament, and the marking of the intersection. Two approaches commonly used for manual angle measuring were implemented. The first approach[9] determines the angle by connecting the nucleosome center point to the respective pixels of the filament touching the nucleosome at its boundary (compare Fig. S6a). The intersection angle $\hat{\theta}$ between the two lines connecting the intersecting points p_1 and $p_2$with the nucleosome center c is calculated by:

$$\hat{\theta} = \arccos \frac{a \cdot b}{||a|| \cdot ||b||}, \tag{1}$$

where $a = c - p_1$ and $b = c - p_2$. This angle can be used in various analysis tasks, but does not represent the opening angle of the nucleosomes, which is why we adopted and automated a technique proposed by Kepert *et al.*[10]. Instead of relying on a single pixel for each branch, a line is fitted to each side. This is done by least square minimization using a first order polynomial for the given branch points, returning a slope m and an offset t for each branch line. Subsequently the corrected angle θ between the two lines is calculated with an updated intersecting point $c = [c_x \, c_y]^\top$ of the two fitted lines given by:

$$c_x = -\frac{t_1 - t_2}{m_1 - m_2}, \qquad c_y = m_1 c_x + t_1 = m_2 c_x + t_2 \, . \tag{2}$$

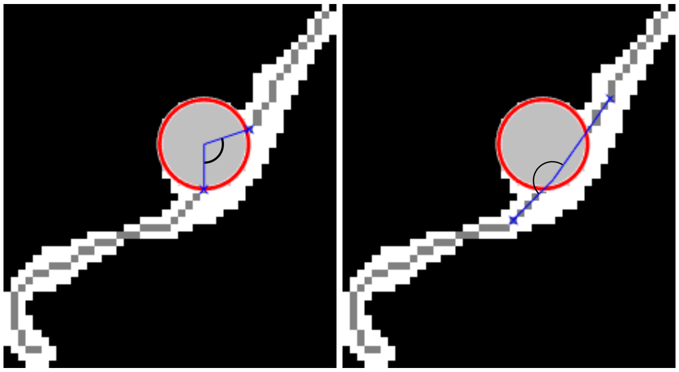

(a) (b)

Figure S6. Angle determination: (a) Estimated angle $\hat{\theta}$ for the method over the radius center, and (b) angle θ determined by our method using line fitting. The results differ significantly for certain cases.

B Performance evaluation of the image analysis method

As demonstrated in Table 1, QuantAFM robustly determines the contour lengths of DNA fragments, resulting in the expected nm/bp ratio of 0.34 with 4% standard deviation. The indicated detection rate was calculated as follows: For 599 bp DNA the automated image analysis method detected 818 objects within a contour length range of 170 nm to 230 nm. 754 of these objects were valid DNA objects and correctly traced, which was verified by visual inspection (92%). 169 valid DNA objects were incorrectly detected and measured. Therefore, when considering all objects (818 +169) and defining the objects which were detected but not valid (64) as well as the undetected objects (169) as errors, the automated image analysis method detected and measured 76% of the assessable objects correctly (detection rate). For 464 bp DNA QuantAFM detected 773 objects within the contour length range from 130 nm to 180 nm. From these, 693 were valid DNA objects (90%). 97 valid DNA objects were found on the images, which results in a detection rate of 80%. However, it is important to note that the performance of automated image analysis strongly depends on image quality, especially for nucleosome data. To obtain a better understanding of the automated image analysis performance, 10 images from wild type nucleosomes (599 bp) were analyzed with a single configuration (one setting for all images). The images included 254 valid mono-nucleosomes (determined by visual inspection categorizing valid objects to be obviously within the expected length range and having no intersections). Without Non-Local Means filtering and background equalization only on 4 images objects were detected within the expected contour length range (130 nm -190 nm). Without visual inspection of the objects, the algorithm returned 56 objects inside the length range, after inspection a mere 36 objects remained. Using QuantAFM with standard configurations, 207 objects in the expected length range were detected. After visual inspection of the detected objects the total number of fully automatically analyzed valid objects was 165, which corresponds to 65% (165 out of 254) detection rate.

C DNA fragments

PCR on the pGEM-3z/601 plasmid using the primers in Table S1 resulted in the 464 bp and 599 bp DNA fragments which both contain the 147 bp long Widom-601 sequence (marked in red). For both fragments, the half from the 5' end to the middle of the fragment corresponds to the α-side and the half of the 3' end to the β-side[11]. Therefore, nucleosomes reconstituted on the 464 bp DNA, have 153 bp and 164 bp protruding DNA arms, where the shorter corresponds to the β-side and the longer to the α-side. For nucleosomes reconstituted on the 599 bp DNA the shorter protruding DNA arm corresponds to the α-side (183 bp) and the longer to the β-side (269 bp).

Primer	Sequence 5'-3'
464 fwd	TAACGCCAGGGTTTTCCCAG
464 rev	CAGCTATGACCATGATTACGCC
599 fwd	GCAAGGCGATTAAGTTGGG
599 rev	CGCAACGCAATTAATGTGAG

Table S1. Primers for PCR on pGEM-3z/601 plasmids.

464 bp fragment

5' TAACGCCAGGGTTTTCCCAGTCACGACGTTGTAAAACGACGGCCAGTGAATTGTAATACGACTCACTATAGGGCGAATTCG
AGCTCGGTACCCGGGGATCCTCTAGAGTCGGAGCTCGGAACACTATCCGACTGGCACCGGCAAGGTCGCTGTTCAATACATG
CACAGGATGTATATATCTGACACGTGCCTGGAGACTAGGGAGTAATCCCCTTGGCGGTTAAAACGCGGGGGACAGCGCGTACG
TGCGTTTAAGCGGTGCTAGAGCTGTCTACGACCAATTGAGCGGCCTCGGCACCGGGATTCTCCAGGGCGGCCGCGTATAGGGT
CCATCACATAAGGGATGAACTCGGTGTGAAGAATCATGCTTTCCTTGGTCATTAGGATCCCGGACCTGCAGGCATGCAAGCTT
GAGTATTCTATAGTGTCACCTAAATAGCTTGGCGTAATCATGGTCATAGCTG

599 bp fragment

5' GCAAGGCGATTAAGTTGGGTAACGCCAGGGTTTTCCCAGTCACGACGTTGTAAAACGACGGCCAGTGAATTGTAATACGAC
TCACTATAGGGCGAATTCGAGCTCGGTACCCGGGGATCCTCTAGAGTCGGAGCTCGGAACACTATCCGACTGGCACCGGCAAG
GTCGCTGTTCAATACATGC ACAGGATGTATATATCTGACACGTGCCTGGAGACTAGGGAGTAATCCCCTTGGCGGTTAAAACG
CGGGGGACAGCGCGTACGTGCGTTTAAGCGGTGCTAGAGCTGTCTACGACCAATTGAGCGGCCTCGGCACCGGGATTCTCCAG
GGCGGCCGCGTATAGGGTCCATCACATAAGGGATGAACTCGGTGTGAAGAATCATGCTTTCCTTGGTCATTAGGATCCCGGAC
CTGCAGGCATGCAAGCTTGAGTATTCTATAGTGTCACCTAAATAGCTTGGCGTAATCATGGTCATAGCTGTTTCCTGTGTGAA
ATTGTTATCCGCTCACAATTCCACACAACATACGAGCCGGAAGCATAAAGTGTAAAGCCTGGGGTGCCTAATGAGTGAGCTAA
CTCACATTAATTGCGTTGCG

D Verification of nucleosome and chromatosome reconstitution and persistence

Reconstitutions were controlled via electrophoretic mobility shift assay (EMSA). Figure S7a shows that nucleosomes have formed, indicated by a reduced electrophoretic mobility compared to free DNA. H2A R81E/R88E mutated (mut) nucleosomes show a more diffuse band than wild type (wt). Nucleosomes with histone H1 showed a decreased electrophoretic mobility indicating for a binding of linker histone to the nucleosomes[12,13]. To further verify the presence of histone H1 after deposition on Poly(L)lysine (PL) coated surface we used Alexa488 labeled histone (H1-T77C). We tested if H1-T77C-Alexa488 fluorescence signal can be detected on samples adsorbed to PL coated surface using the same experimental conditions as for the AFM experiments (Fig. S7b). Therefore, circular glass slides were coated with PL or incubated only with buffer, washed and dried. Afterwards, the sample (either reconstituted chromatosomes with H1-T77C-Alexa488, or free H1-T77C-Alexa488) was adsorbed to the center spot of the glass slide, washed, dried and fluorescent signal was detected with Typhoon multimode imager (GE Healthcare, Germany). Figure S7b i) and ii) shows that the fluorescence signal of free H1-T77C-Alexa488 (50 nM concentration) can be detected on the glass surface with and without PL coating. The signal intensity is comparable on both surfaces. In contrast, the signal of H1-T77C-Alexa488 bound to mutated nucleosomes (5 nM concentration) is clearly higher on PL coated glass surface (Fig. S7b) (iii) than on glass surface without PL coating (iv). The red arrows in (Fig. S7b) mark unavoidable background noise (dust contaminations) resulting in dotted signals which can be seen on the sample surfaces, the edges and even beyond the coverslip surface. However, the results of this experiment suggests that histone H1 bound to nucleosomes are absorbed to PL coated surface after experimental procedure. This clearly indicates that the found structural differences detected via AFM are related to histone H1 bound to the nucleosomes.

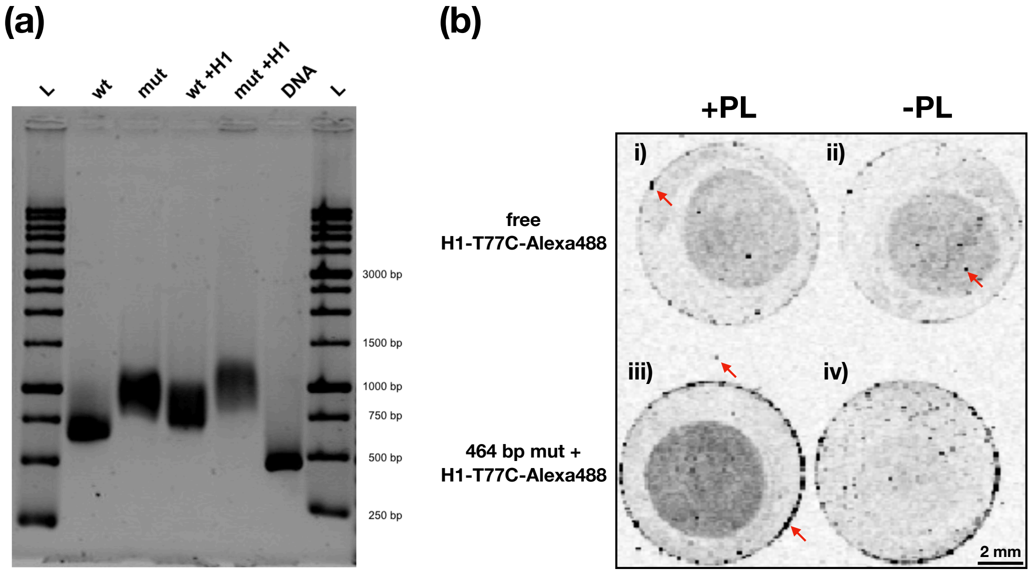

Chromatosome reconstitution and persistence

Figure S7. Nucleosome reconstitution and linker histone binding was controlled via EMSA and fluorescently labeled histone H1. (a) EMSA of nucleosomes reconstituted on 464 bp DNA. Nucleosome samples (wt or mut) with or without histone H1 (+H1), free 464 bp DNA fragments (DNA), as well as Bioron 1 kb DNA ladder (L) run in 1% agarose gel in TBE buffer (pH 7.5). Nucleosome samples are clearly separated from band of free DNA. Changed electrophoretic mobility can be seen upon mutation and presence of histone H1. (b) circular glass slides (i-iv) were imaged with Typhoon multimode imager at 488 nm excitation wavelength and BP 520/40 nm emission filter with 100 μm resolution. Free histone H1-T77C-Alexa488 i), ii) and mutated (mut) 464 bp nucleosomes + histone H1-T77C-Alexa488 iii), iv) were diluted in (10mM Tris-HCl, 0.1 mM EDTA, 15 mM NaCl, pH 7.5) buffer to nM concentrations (50 nM for free H1-T77C-Alexa488, 5 nM for mut chromatosomes). 15 μl of the solution was deposited on the center of the slides either on PL coated surface (+PL, same procedure as for AFM experiments) or on glass slides without PL coating (-PL), washed and dried (see Methods Section AFM experiments). i) free H1-T77C-Alexa488 on PL coated glass slides, ii) free H1-T77C-Alexa488 on non-coated glass slides iii) 464 bp mut H1-T77C-Alexa488 on PL coated glass slides, iv) 464 bp mut H1-T77C-Alexa488 on non-coated glass slides. Red arrows indicate exemplary contaminations, which appear as dotted signal on the image.

E Mutated 464 bp nucleosomes with and without histone H1

Supplementary Fig. S8 shows representative AFM images as well as scatter plots of mutated 464 bp nucleosomes without and with histone H1.

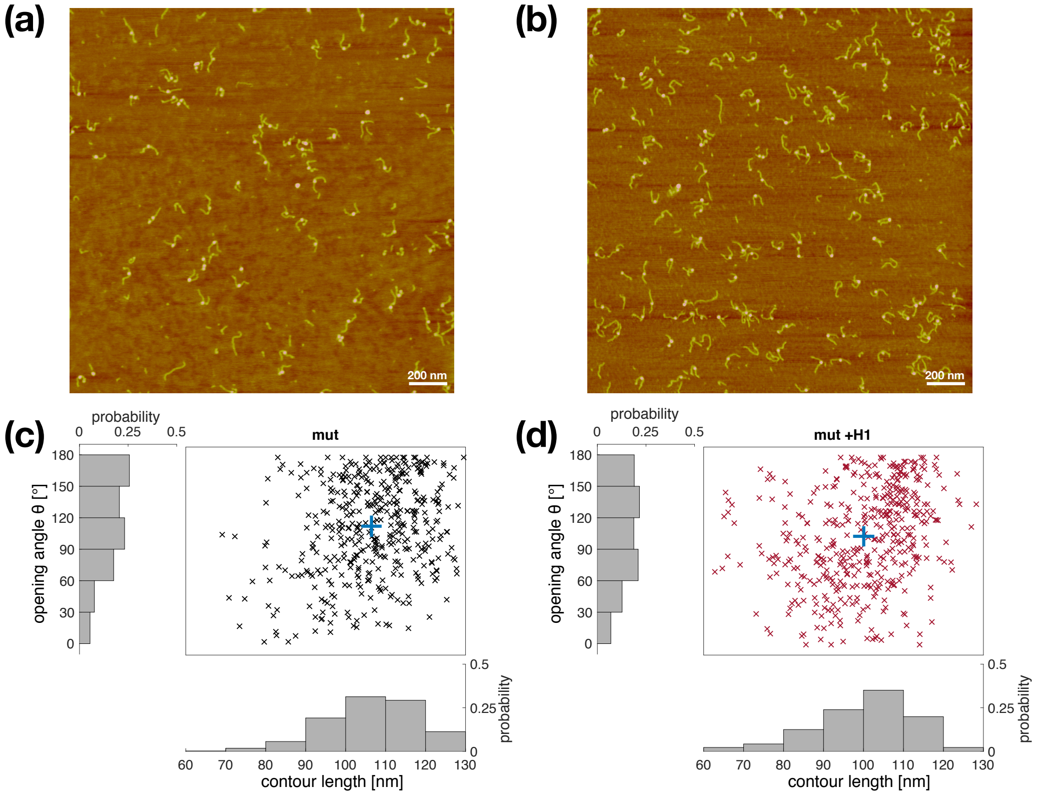

Mutated nucleosomes without and with histone H1

Figure S8. (a) and (b) show example images of mutated nucleosomes reconstituted on 464 bp DNA, without and with histone H1, respectively. (c) and (d) show scatter plots of the opening angle and the sum of the protruding DNA arm contour length of mutated nucleosomes (c) without ($n = 444$, black) and (d) with histone H1 ($n = 448$, red); centroids are highlighted in blue. Histograms present normalized distributions for opening angle θ (30° bin width) as well as for protruding DNA arm contour length (bin width 10 nm).

F Parameters of 599 bp nucleosome objects show the same trends as for 464 bp nucleosome objects

Experiments with 599 bp nucleosomes resulted in same trends as for 464 bp nucleosomes, for all discussed parameters. Figure S9a shows a representative zoom of an image with nucleosomes reconstituted on 599 bp DNA. EMSA of 599 nucleosome samples (Fig. S9b) show a shift in electrophoretic mobility upon mutation and histone H1. The automatically extracted parameters (Fig. S9c) show that the trends for the contour length parameters of 599 bp nucleosome objects are similar as for the 464 bp nucleosome objects (unbound DNA contour length : wt +H1 < wt < mut +H1< mut). Furthermore, also for the opening angle θ the main observations as for the 464 bp nucleosome objects hold.

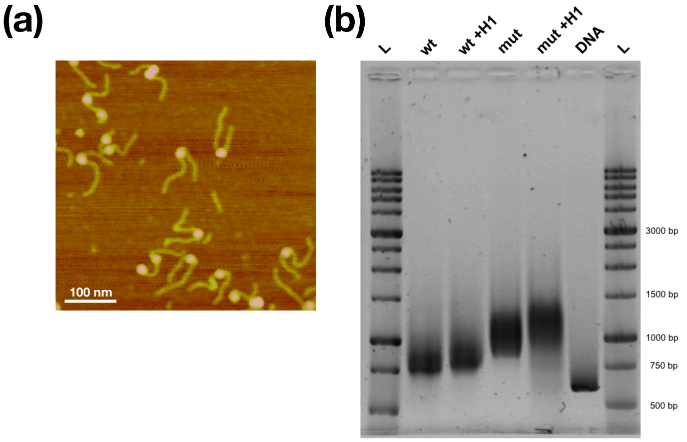

(c)

parameter	opening angle θ mean ± sd [°]	radius mean ± sd [nm]	short DNA arm mean ± sd [nm]	long DNA arm mean ± sd [nm]
wt 599 (n = 175)	73.6 ± 40.6	10.3 ± 1.5	57.2 ± 8.2	85.2 ± 9.0
wt +H1 599 (n = 188)	76.8 ± 37.9	10.9 ± 1.5	55.2 ± 9.6	81.3 ± 10.3
mut 599 (n =184)	110.5 ± 46.3	10.4 ± 1.5	61.2 ± 9.0	85.9 ± 8.9
mut +H1 599 (n = 168)	102.9 ± 50.6	10.5 ± 1.5	60.0 ± 10.1	82.4 ± 8.6

599 bp nucleosomes

Figure S9. (a) Enlarged section of AFM image of wt nucleosomes reconstituted on 599 bp DNA, indicating a clearly different DNA arm contour length. (b) EMSA of nucleosomes reconstituted on 599 bp DNA. Nucleosome samples (wt or mut) with or without histone H1 (+H1), free 599 bp DNA fragments (DNA), as well as Bioron 1 kb DNA ladder (L) run in 1% agarose gel in TBE buffer. Nucleosome samples are clearly separated from the band of free DNA. Changed electrophoretic mobility can be seen upon mutation and presence of histone H1. (c) Mean values with standard deviation of extracted structural parameters of automatically analyzed 599 bp nucleosome images. Parameters were DNA opening angle θ, radius of the nucleosome object, contour length of short protruding DNA arm, and contour length of long protruding DNA arm. Mean values show the same trend as for 464 bp nucleosomes (see Table 2).

References

1. Buades, A., Coll, B. & Morel, J.-M. Non-Local Means Denoising. *Image Processing On Line* **1**, 208–212, DOI: 10.5201/ipol.2011.bcm_nlm (2011).
2. Sundstrom, A. *et al.* Image Analysis and Length Estimation of Biomolecules Using AFM. *IEEE Transactions on Inf. Technol. Biomed.* **16**, 1200–1207, DOI: 10.1109/TITB.2012.2206819 (2012).
3. Duda, R. O. & Hart, P. E. Use of the Hough transformation to detect lines and curves in pictures. *Commun. ACM* **15**, 11–15, DOI: 10.1145/361237.361242 (1972).

4. Otsu, N. A threshold selection method from gray-level histograms. *IEEE Transactions on Syst. Man, Cybern.* **9**, 62–66, DOI: 10.1109/TSMC.1979.4310076 (1979).
5. Hilditch, C. J. Linear Skeletons From Square Cupboards. In Meltzer, B. & Michie, D. (eds.) *Machine Intelligence 4* (Edinburgh University Press, 1969).
6. Zhang, T. & Suen, C. Y. A fast parallel algorithm for thinning digital patterns. *Commun. ACM,* **27(3)**, 236–239, (1984).
7. Rivetti, C. & Codeluppi, S. Accurate length determination of DNA molecules visualized by atomic force microscopy: evidence for a partial B- to A-form transition on mica. *Ultramicroscopy* **87**, 55–66 (2001).
8. Kulpa, Z. Area and perimeter measurement of blobs in discrete binary pictures. *Comput. Graph. Image Process.* **6**, 434–451, DOI: https://doi.org/10.1016/S0146-664X(77)80021-X (1977).
9. Bussiek, M., Toth, K., Brun, N. & Langowski, J. DNA-loop formation on nucleosomes shown by in situ scanning force microscopy of supercoiled DNA. *J Mol Biol* **345**, 695–706, DOI: 10.1016/j.jmb.2004.11.016 (2005).
10. Kepert, J. F. *et al.* Conformation of reconstituted mononucleosomes and effect of linker histone H1 binding studied by scanning force microscopy. *Biophys J* **85**, 4012–4022, DOI: 10.1016/s0006-3495(03)74815-2 (2003).
11. Gansen, A. *et al.* High precision FRET studies reveal reversible transitions in nucleosomes between microseconds and minutes. *Nat. Commun.* **9**, 4628, DOI: 10.1038/s41467-018-06758-1 (2018).
12. Bussiek, M., Toth, K., Schwarz, N. & Langowski, J. Trinucleosome compaction studied by fluorescence energy transfer and scanning force microscopy. *Biochemistry* **45**, 10838–10846, DOI: 10.1021/bi060807p (2006).
13. Shukla, M. S. *et al.* The docking domain of histone H2A is required for H1 binding and RSC-mediated nucleosome remodeling. *Nucleic Acids Res* **39**, 2559–2570, DOI: 10.1093/nar/gkq1174 (2011).
